# Biometry and volumetry in multi-centric fetal brain magnetic resonance imaging: assessing the bias of super-resolution reconstruction

**DOI:** 10.1007/s00247-025-06347-7

**Published:** 2025-08-08

**Authors:** Thomas Sanchez, Angeline Mihailov, Mériam Koob, Nadine Girard, Aurélie Manchon, Ignacio Valenzuela, Marta Gómez-Chiari, Gerard Martí Juan, Alexandre Pron, Elisenda Eixarch, Gemma Piella, Miguel Angel Gonzalez Ballester, Oscar Camara, Vincent Dunet, Guillaume Auzias, Meritxell Bach Cuadra

**Affiliations:** 1 University of Lausanne, Lausanne, Switzerland; 2 Lausanne University Hospital (CHUV), Rue du Bugnon 46, Lausanne, 1011 Switzerland; 3 Institut de Neurosciences de la Timone, Marseille, France; 4 Hôpital de la Timone, Marseille, France; 5 BCNatal|Fetal Medicine Research Center (Hospital Clínic and Hospital Sant Joan de Déu, Universitat de Barcelona), Barcelona, Spain; 6 Institut d’Investigacions Biomèdiques August Pi i Sunyer (IDIBAPS), Barcelona, Spain; 7 Centre for Biomedical Research on Rare Diseases (CIBERER), Barcelona, Spain; 8 Hospital Sant Joan de Déu Barcelona, Barcelona, Spain; 9 Pompeu Fabra University, Barcelona, Spain; 10 CIBM Center for Biomedical Imaging, Lausanne, Switzerland

**Keywords:** Bias, Biometry, Fetal brain MRI, Reproducibility, Super-resolution reconstruction

## Abstract

**Background:**

Fetal brain MRI is increasingly used to complement ultrasound imaging. Images are processed using complex super-resolution reconstruction pipelines, which may bias biometric and volumetric measurements.

**Objective:**

To assess the consistency of 2-dimensional (D) biometric and 3-D volumetric measurements across three hospitals using three widely used super-resolution reconstruction pipelines.

**Materials and methods:**

This retrospective multi-centric study used T2-weighted fetal brain MRI scans acquired at three hospitals between 2009 and 2023. MRIs from each subject were reconstructed with each super-resolution reconstruction method, and biometric measurements were performed by four experts. Automated 3-D volumetry was performed using a state-of-the-art segmentation method. A qualitative evaluation assessed the clinicians’ likelihood of using super-resolution reconstructed volumes in their practice.

**Results:**

Eighty-four healthy subjects were included. Biometric measurements revealed statistically significant changes that consistently remained below voxel width (0.8 mm; *P*<0.001). Automated 3-D volumetry revealed small systematic effects (<2.8%; *P*<0.001). The qualitative evaluation showed systematic differences between super-resolution reconstruction methods for the perception of white matter intensity (*P*=0.02) and sharpness of the image (*P*=0.01).

**Conclusion:**

Variations in 2-D and 3-D quantitative measurements did not show any large systematic bias when using different super-resolution reconstruction methods for clinical radiological assessment across centers, scanners, and raters.

**Supplementary Information:**

The online version contains supplementary material available at 10.1007/s00247-025-06347-7.

## Introduction

Fetal brain magnetic resonance imaging (MRI) is increasingly used as a complement to ultrasound (US) imaging for confirming or ruling out equivocal findings [1]. Its excellent soft tissue contrast and image resolution enables more accurate measurements of the fetal brain as well as a better parenchymal signal, critical for detecting cortical malformations and subtle white matter anomalies [2].

Antenatal brain MRI routine assessment combines qualitative morphological evaluation and biometric measurements. In routine clinical practice, fetal brain MRI biometry is performed on T2-weighted (T2w) stacks of two-dimensional slices with 2–5 mm thickness and 0.5–1 mm in-plane resolution, usually acquired following three orthogonal planes. However, fetal and maternal motion can lead to oblique acquisition planes, which, combined with the anisotropic image resolution, can make it difficult to carry out precise biometric measurements. Although some studies have compared measurements done on MRI to US reference values [3–7] used to establish deviation from normality, MRI-based biometric measurements are still not recommended in clinical practice because of the challenge of acquiring a precise slice orientation with MRI.

In the past decade, super-resolution reconstruction methods [8–14] have emerged, allowing the combination of motion-corrupted, low-resolution T2w series into a high-resolution 3-dimensional (D) isotropic volume. These 3-D volumes are valuable for fetal brain biometry, since they enable flexible navigation in any plane, facilitating the selection of optimal planes for precise biometric measurements [15–17]. Moreover, they enable a volumetric (3-D) analysis, supported by several automated pipelines [8, 10, 12–14, 18, 19]. These techniques pave the road towards a more accurate characterization of normal and pathological fetal neurodevelopment using MRI.

Early works on super-resolution reconstruction 3-D volumes have compared the consistency of their biometric measurements with those from US and low-resolution slices [16, 20–22]. Kyriakopoulou et al. [16] used super-resolution reconstructed volumes reconstructed using the Slice-to-Volume Reconstruction method [8, 10] to build normative models of both biometric and volumetric structures. Khawam et al. [20] studied the inter-rater reliability between biometric measurements on T2w series and volumes reconstructed using the Medical Image Analysis Laboratory Super-Resolution ToolKit (MIALSRTK) [12, 19], while Lamon et al. [21] focused on corpus callosum biometry, comparing US, T2w, and super-resolution reconstructed volumes reconstructed using MIALSTRK [12, 19]. However, these works relied on a single super-resolution reconstruction method; thus, its replication with other super-resolution reconstruction methods remains to be proven. Recently, Ciceri et al. [22] compared for the first time 2-D biometry across multiple super-resolution reconstruction methods (MIALSRTK [12, 19], NiftyMIC [13], and the Slice-to-Volume Reconstruction ToolKit (SVRTK) [10, 18]), focusing on the 20–21 gestational weeks period. They showed that MIALSRTK and NiftyMIC achieved a good reconstruction success rate and were consistent with T2w series measurements, while SVRTK showed many failed reconstructions and was excluded.

However, these works were all limited to mono-centric data, and did not consider whether super-resolution reconstruction methods could improve inter-rater reliability or if they introduced systematic biases in quantitative measurements. Ciceri et al. [22] did not disentangle the effect of data quality from the impact of the super-resolution reconstruction algorithm. By conflating the success rate of the compared super-resolution reconstruction methods and the quality of the biometric measurements, they could not answer the following question: when different super-resolution reconstruction methods yield good quality results, will the biometric measurement values remain consistent? Or, framed differently: does the reconstruction process of any super-resolution reconstruction method introduce alterations that systematically bias the biometric evaluation, even when the super-resolution reconstruction is of good quality?

We hypothesized that given high-quality reconstructions, 2-D and 3-D measurements would be consistent across different super-resolution reconstruction methods, but that experts would remain cautious about using super-resolution reconstructions for clinical assessments, because of alterations in the intensity of the reconstructed image. The purpose of this study was to evaluate the clinical usefulness of super-resolution reconstruction and assess whether these methods could introduce artifacts that would systematically bias measurements taken from the reconstructed volumes.

## Materials and methods

### Dataset

#### Population

**Fig. 1:**
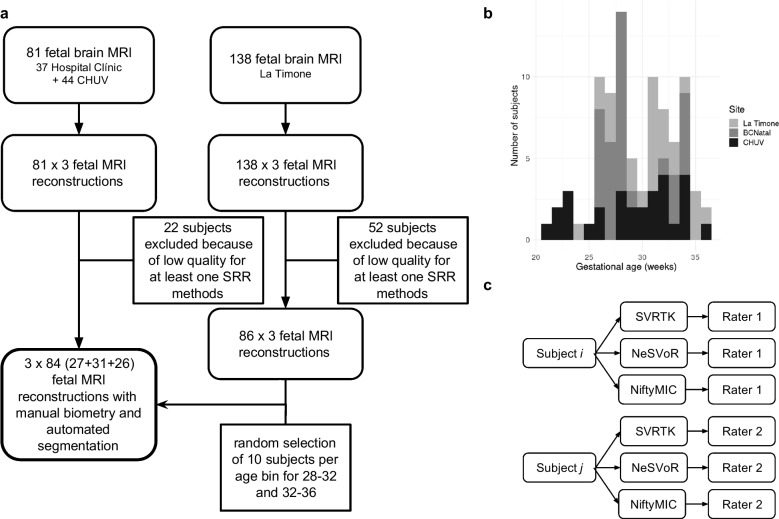
General description of the study. **a** Flowchart of our study sample shows inclusion and exclusion. There was a total of 219 pregnant patients who were imaged across three centers. Seventy-four magnetic resonance imaging (MRI) examinations were excluded due to poor-quality reconstruction, resulting in 145 MRI examinations that were annotated and automatically segmented. After selection of subjects in relevant age bins, this resulted in 84 MRI examinations analyzed (27 for ages [21–28), 31 for [28,32), and 26 for [32–36)). **b** Distribution of gestational ages across the different sites. **c** Design of the study. The subjects are nested within the raters. The raters considered the subjects from their center (M.K. for CHUV, I.V. for Hospital Clínic and N.G., A.Ma. for La Timone) and performed the measurements on every reconstruction for each subject. *NeSVoR*, Neural Slice-to-Volume Reconstruction; *SVRTK*, Slice-to-Volume Reconstruction ToolKit

Brain MRI examinations were retrospectively collected from ongoing research studies at the three hospitals: Lausanne University Hospital (H1; CHUV, Lausanne, Switzerland), Hospital Clínic de Barcelona (H2; Barcelona, Spain) and La Timone (H3; Marseilles, France). Exclusion criteria included twin pregnancies and any pathology or malformation in the fetal MRI scans. The study received ethical approval from each center’s institutional review board (CHUV: CER-VD 2021-00124, Hospital Clínic: HCB/2022/0533, La Timone: Aix-Marseille University N°2022-04-14-003). Fetal examinations were equally distributed across three gestational age (GA) bins representing different stages of fetal brain development: [21, 28) weeks, [28, 32) weeks, and [32, 36) weeks. A flow diagram of included and excluded MRI examinations is shown in Fig. 1.

#### Data

Fetal MRI data were acquired with different Siemens Healthineers scanners (Erlangen, Germany) at 1.5 tesla (T) or 3 T across hospitals. The fetal brain MRI protocol included T2w HASTE (half-Fourier acquisition single-shot turbo spin echo imaging) sequences acquired in three orthogonal directions (axial, coronal, sagittal). Details on the different MRI acquisition parameters and number of acquisitions per subject are available in Table 1. There is some degree of heterogeneity in the acquisition, which reflects the variations in clinical practice, as acquisition protocols can vary across imaging centers [1].

**Table 1:** Metadata regarding the acquisition parameters, the gestational ages of participants, the resolution of the T2w series, and the number stacks used in the reconstruction algorithm

Site	Scanner	Field [T]	*n*_sub_	21–28	28–32	32–36	Low-resolution [mm^3^]	*n*_stacks_
**CHUV**	Aera	1.5	19	9	8	2	1.12 × 1.12 × 3.3	6.2 ± 3.1
	MAGNETOM Sola	1.5	8	0	3	5	1.12 × 1.12 × 3.3	6.3 ± 1.3
**Hospital Clínic**	Skyra	3.0	2	0	0	2	0.55 × 0.55 × 3.0	5.5 ± 2.1
	Aera	1.5	29	12	10	7	0.55 × 0.55 × 2.8	12.0 ± 3.3
**La Timone**	SymphonyTim	1.5	17	3	6	8	0.74 × 0.74 × 3.5	4.7 ± 1.8
	Skyra	3.0	9	3	4	2	0.68 × 0.68 × 3.0	3.4 ± 0.7

n_sub_, number of subjects

#### Data processing

As clinical fetal brain MRI acquisitions feature anisotropic resolution, the data acquired in different orientations are reconstructed into a single, high-resolution volume through super-resolution reconstruction methods. Each subject was reconstructed using three widely used super-resolution reconstruction toolkits: Neural Slice-to-Volume Reconstruction (NeSVoR) (v.0.5.0) [14], NiftyMIC (v.0.9.0) [13], and SVRTK (v.auto-2.2.0) [10]. These pipelines were chosen as they are widely used in the community and are representative of both classical inverse problem approaches [13, 18] and self-supervised deep learning-based reconstruction methods [14]. Depending on the hospital, stacks with high levels of motion or signal drops were excluded through visual inspection [20] and/or automated quality control [23]. At La Timone and Hospital Clínic, stacks were processed with non-local means denoising [24] and N4 bias field correction [25]. Each subject was then reconstructed using the default parameters of the three super-resolution reconstruction methods, at 0.8 mm isotropic resolution. The resulting super-resolution reconstructed volumes were aligned to a standard orientation.

For poor-quality reconstructions, different stack combinations were tested until the image quality was deemed sufficient by visual assessment (no evident artifacts or errors from registration/reconstruction). If no combination resulted in a sufficiently high-quality reconstruction, the subject was excluded from the study.

#### Biometric measurements

**Fig. 2:**
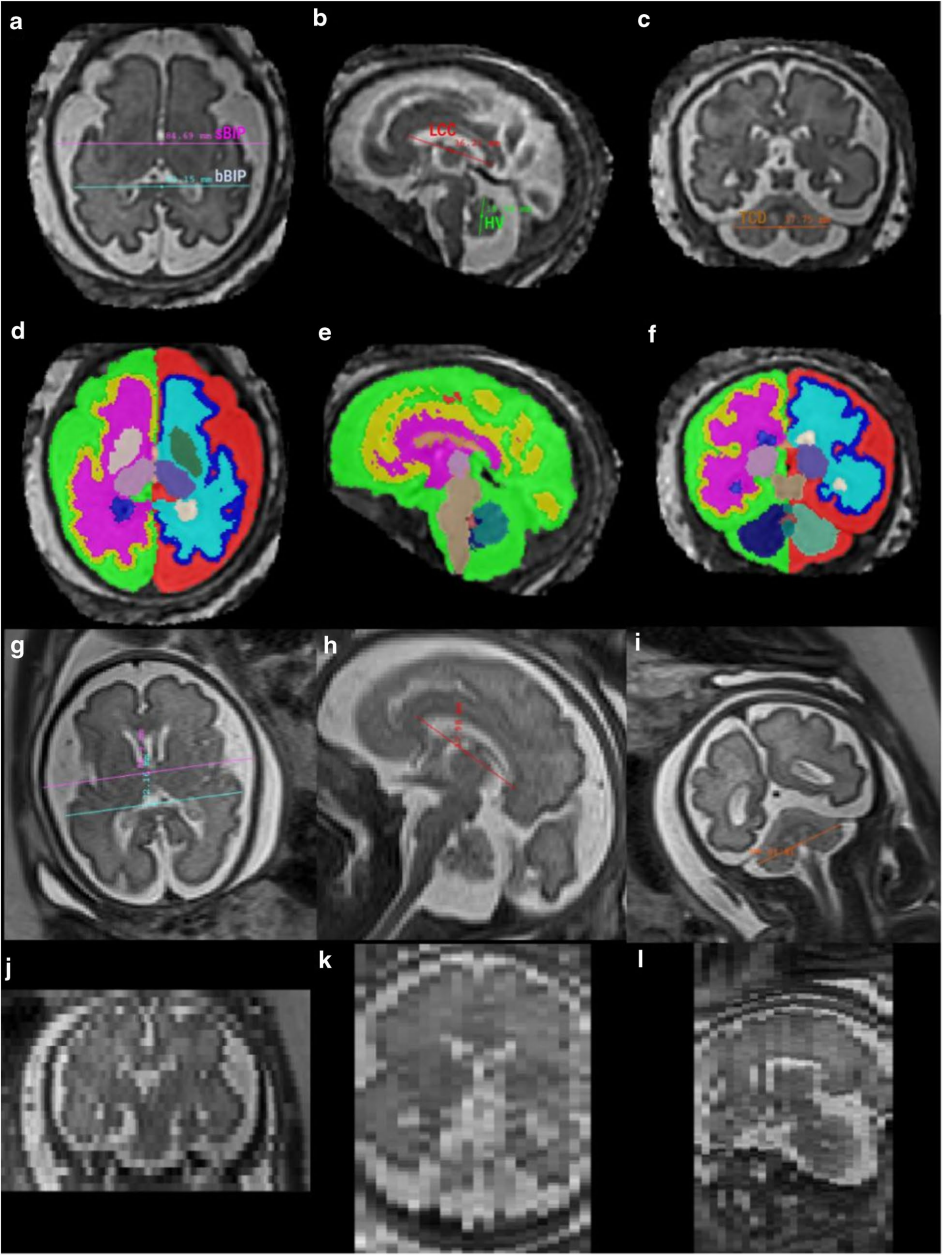
Illustration of measurements on a 31-week-old healthy subject using T2w HASTE data. **a**–**c** Biometric measurements on a volume reconstructed using the Slice-to-Volume Reconstruction ToolKit (SVRTK). Axial (**a**): brain and skull biparietal diameters (bBIP and sBIP). Sagittal (**b**): length of the corpus callosum (LCC) and height of the vermis (HV). Coronal (**c**): transverse cerebellar diameter (TCD). **d**–**f** Automated segmentation using the Brain vOlumetry and aUtomated parcellatioN (BOUNTI) method in axial (**d**), sagittal (**e**), and coronal (**f**) planes. **g**–**i** Measurements on the T2w HASTE stacks in axial (**g**), sagittal (**h**), and coronal (**i**) planes. Each *column* represents a different stack. The stacks were re-oriented for visualization purposes. **j**–**l** Through-plane view of the low-resolution images of images (**g**, **h**, **i**), showing the thick slices of the low-resolution acquisitions in coronal (**j**), axial (**k**), and sagittal (**l**) planes. *bBIP*, brain biparietal diameters; *HV*, height of the vermis; *LCC*, length of the corpus callosum; *sBIP*, skull biparietal diameters; *TCD*, transverse cerebellar diameter

Biometric measurements were performed on both low-resolution 2-D stacks and 3-D super-resolution reconstructed volumes using ITK-SNAP (University of Pennsylvania, PA, USA). Measures were performed on each site by medical experts in obstetrics and/or pediatric image analysis: M.K. (15 years of experience) for CHUV, I.V. (5 years of experience) for Hospital Clínic and N.G (> 20 years of experience), and A. Ma. (5 years of experience) for La Timone. This resulted in a design where subjects are nested within the raters (Fig. 1). Following established guidelines for fetal brain MRI biometry [1, 3, 16, 26], the following measurements were performed: length of the corpus callosum (LCC), height of the vermis (HV), brain and skull biparietal diameters (bBIP, sBIP), and transverse cerebellar diameter (TCD). An example of the measurements on a subject is shown in Fig. 2. These measurements were then compared to the reference values obtained by Kyriakopoulou et al. [16].

On the low-resolution stacks, each rater chose the stack best suited (in terms of alignment and image quality) for each measurement. On the 3-D super-resolution reconstructed volumes, raters had the option to re-align (manual rigid transformation) the images prior to performing the measurements. In total, the four different raters each performed around 550 measurements (5 structures × 4 variants (1 low-resolution + 3 super-resolution reconstructions) × 26–29 subjects).

#### Automated volumetry

Automated volumetric evaluation was carried out on the super-resolution reconstructed volumes using Brain vOlumetry and aUtomated parcellatioN (BOUNTI) [27], a recent deep learning segmentation method. BOUNTI segments the brain into 19 different regions and was trained on a large corpus of manually segmented brains volumes. An illustration of the segmentations is provided in Fig. 2. In our analysis, we considered five volumetric measurements for which reference values are available [16]: extra-cerebral cerebrospinal fluid (extra-cerebral CSF), cortical gray matter (cortical GM), cerebellum, supratentorial brain tissue (ST), and total lateral ventricles. Cortical GM and cerebellum measurements were also compared to the growth curves from Machado-Rivas et al. [28], which used the methods of Kainz et al. [11] to reconstruct the T2w stacks, and automated segmentation with an atlas-based approach [15].

#### Qualitative assessment

We aimed at obtaining expert feedback on the appearance, particularly on the aspects of intensity and visibility, of key anatomical structures used to assess fetal development. Four neuroradiologists (N.G., > 20 years of experience; A.Ma., 5 years of experience; M.K., 15 years of experience; M.G.C., 12 years of experience) were asked to qualitatively assess the volumes reconstructed from six subjects using all three super-resolution reconstruction methods considered. The subjects were selected to represent different GA bins (26, 28, 29, 30, 32, and 34 weeks) with high-quality 3-D super-resolution reconstructed volumes for all subjects and methods to avoid any bias. In a first round of evaluation, the clinicians visualized all super-resolution reconstructed volumes from a given subject and were asked to assess how clearly different structures appeared in the super-resolution reconstructed volume. The details of the questions asked and structures rated are available in supplementary Table S9. In a second stage, raters were asked to compare the super-resolution reconstructed volumes from each subject with the corresponding low-resolution stacks of images. They were first asked to rank the three super-resolution reconstructed volumes for each subject based on their likelihood of use (with ties allowed). They were then asked to determine whether they would choose the super-resolution reconstructed volume over the low-resolution stacks for their clinical assessment, and whether the super-resolution reconstructed volume provided more information than the low-resolution stacks for a radiological evaluation.

### Statistical analysis

A univariate analysis was initially carried out to assess the influence of the super-resolution reconstruction algorithm on the biometric (respectively volumetric) measurements. Due to the non-Gaussian distribution of the data, a Friedman test (the non-parametric equivalent of a repeated measures ANOVA, *N*=252, degrees of freedom =2) was used to test the difference across super-resolution reconstruction methods. We did not apply corrections for multiple comparisons to detect even small statistical effects related to the super-resolution reconstruction techniques, as correction would make it easier to support our hypothesis. Post-hoc testing was done using pairwise Wilcoxon rank-sum tests, and Bonferroni correction for multiple comparisons was applied at this stage. Effect sizes were reported as $Z/\sqrt{N}$.

We confirmed these results using multivariate regression to evaluate the impact of super-resolution reconstruction on biometric (resp. volumetric) measurements while accounting for covariates. A t-distributed Generalized Additive Model for Scale and Location (GAMLSS) [29, 30] was fitted with the biometric (resp. volumetric) measurement as the response, the super-resolution reconstruction algorithm as the fixed effect of interest, gestational age (GA) as a covariate, rater as a covariate for the biometry only (as the volumetry is computed automatically), and subject as a random effect.

The choice of a GAMLSS model over a simpler t-distributed linear mixed effect (LME) model was based on visual inspection of the residual distribution (R function fitdistrplus::descdist) and of the cumulative distribution function (R function DHARMa::simulateResiduals). While both the LME and the GAMLSS had a well-aligned cumulative distribution function, the GAMLSS model showed a less dispersed residual distribution, suggesting more stable estimates.

The qualitative analysis relied on a smaller sample. We nonetheless carried out a univariate analysis using a Friedman test (*N*=72, degrees of freedom =2). When significant results were found, post-hoc analysis testing was done using pairwise Wilcoxon rank-sum tests, with Bonferroni correction for multiple comparisons. All statistical analyses were carried out using the R software version 4.2.2 (R Foundation for Statistical Computing, Vienna, Austria). To facilitate the analysis of the results, the ratings of R3 were used in a confirmatory analysis as part of a supplementary experiment. The analysis then simply has subjects nested within raters.

## Results

### Population

After application of the inclusion and exclusion criteria (Fig. 1), 252 super-resolution reconstruction from 84 healthy fetuses were included: 29 at CHUV, 29 at Hospital Clínic, and 26 at La Timone. The distribution of gestational age is shown in Fig. 1 and broken down by age bins in Table 1.

### Biometry measurements across super-resolution reconstruction methods

Univariate and multivariate statistics are reported in Table 2. There was no significant difference induced by super-resolution reconstruction methods on LCC and HV in the univariate analysis, very small effects in the multivariate analysis, –0.2±0.06 mm (*P*<0.001) for the NeSVoR-NiftyMIC difference in LCC, and −0.09±0.94 (*P*<0.05) for the NeSVoR-SVRTK difference in HV. When comparisons yielded statistically significant results, the effect sizes systematically remained small (at most 0.43±0.06 mm for the sBIP), smaller than a 0.1% variation and below the width of a voxel (0.8 mm).

**Table 2:** Statistical analyses for biometry measurements. Univariate biometry analysis (*N*=252, df=2) and multivariate biometry analysis using a t-distributed Generalized Additive Model for Scale and Location (GAMLSS) model

	Univariate analysis					Multivariate analysis				
	Friedman		Post-hoc testing			Super-resolution reconstruction effect			Rater effect	
	*P*-value	Comp.	*P*-value	Eff. size	Median diff. [mm]	Est’d effect	*P*-value	Comp	Est’d effect	*P*-value
**LCC**	**0.03**	NeSVoR vs NiftyMIC	>0.05	–	–	−0.21±0.06	**10**^**–4**^	I.V. vs M.K.	1.09±0.05	**10**^**–16**^
		NeSVoR vs SVRTK	>0.05	–	–	0.07±0.05	0.17	I.V. vs N.G.	0.29±0.06	**10**^**–6**^
**HV**	0.92	NeSVoR vs NiftyMIC	–	–	–	−0.03±0.04	0.50	I.V. vs M.K.	−0.23±0.04	**10**^**–7**^
		NeSVoR vs SVRTK	–	–	–	−0.09±0.94	**0.04**	I.V. vs N.G.	−1.03±0.04	**10**^**–16**^
**bBIP**	**9.8 × 10**^**–3**^	NeSVoR vs NiftyMIC	>0.05	–	–	−0.31±0.06	**10**^**–6**^	I.V. vs M.K.	0.57±0.06	**10**^**–16**^
		NeSVoR vs SVRTK	0.03	0.28	−0.3[−3.1,2.4]	−0.42±0.06	**10**^**–11**^	I.V. vs M.K.	−1.20±0.06	**10**^**–16**^
**sBIP**	**6.9 × 10**^**–4**^	NeSVoR vs NiftyMIC	0.01	−0.32	0.4[−1.1,1.9]	0.43±0.06	**10**^**–12**^	I.V. vs N.G.	1.55±0.06	**10**^**–16**^
		NeSVoR vs SVRTK	10^–3^	−0.43	0.4[−1.5,2.3]	0.43±0.05	**10**^**–13**^	I.V. vs M.K.	0.14±0.05	**0.01**
**TCD**	**3.5 × 10**^**–3**^	NeSVoR vs NiftyMIC	0.02	0.30	−0.4[−1.6,0.9]	−0.34±0.04	**10**^**–12**^	I.V. vs N.G.	0.71±0.04	**10**^**–16**^
		NeSVoR vs SVRTK	10^–3^	0.38	−0.3[−1.2,0.9]	−0.38±0.04	**10**^**–14**^	I.V. vs N.G.	−0.70±0.04	**10**^**–16**^

*bBIP* brain biparietal diameters, *HV* height of the vermis, *LCC* length of the corpus callosum, *NeSVoR* Neural Slice-to-Volume Reconstruction, *sBIP* skull biparietal diameters, *SVRTK* Slice-to-Volume Reconstruction ToolKit, *TCD* transverse cerebellar diameter

The multivariate analysis also allowed estimating effects related to the raters, which were consistently larger than the super-resolution reconstruction effects, but remained small. The effect was at most 1.55 mm for sBIP (2.5% variability). These results were confirmed by an additional, single-site analysis, where two raters annotated the same data (see Supplementary materials).

**Fig. 3:**
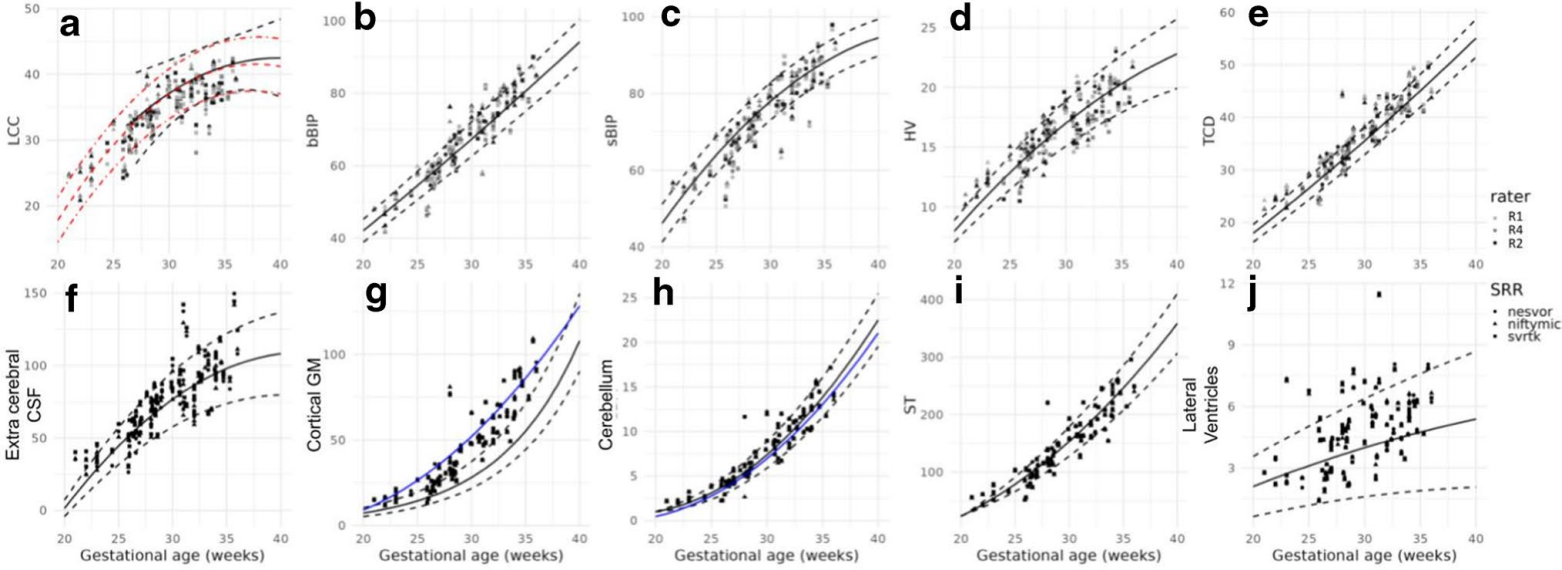
Measurements as a function of gestational age. **a**–**e** Biometric measurements of length of the corpus callosum (**a**), brain biparietal diameter (**b**), skull biparietal diameter (**c**), height of the vermi**s (d**), and transverse cerebellar diameter (**e**) as a function of gestational age, for the different super-resolution reconstruction methods and raters. The *curves* and *dashed lines* represent normative 5^th^, 50^th^, and 95^th^ centiles from Kyriakopoulou et al. [16], except for the length of the corpus callosum (LCC), where the *black curve* is from measurements on HASTE acquisitions from Tilea et al. [3] and the *red* one from ultrasound measurements done by Pashaj et al. [31]. **f**–**j** Volumetric measures of extra-cerebral cerebrospinal fluid (**f**), cortical gray matter (**g**), cerebellum (**h**), supratentorial tissue (**i**), and lateral ventricles (**j**) as a function of gestational age, for the different super-resolution reconstruction methods and sites. The *curves* and *dashed lines* represent normative 5^th^, 50^th^, and 95^th^ centiles from Kyriakopoulou et al. [16] and additional *blue curves* are taken from Machado-Rivas et al. [28]. *bBIP*, brain biparietal diameters; *CSF*, cerebrospinal fluid; *GM*, gray matter; *HV*, height of the vermis, *LCC*, length of the corpus callosum; *NeSVoR*, Neural Slice-to-Volume Reconstruction; *sBIP*, skull biparietal diameters; *ST*, supratentorial brain tissue; *SVRTK*, Slice-to-Volume Reconstruction ToolKit; *TCD*, transverse cerebellar diameter

Growth charts are provided in Fig. 3 (top row) and in line with the centiles estimated in previous works [3, 16, 31]. Further illustration of the different growth curves for the different raters and super-resolution reconstruction is provided in Supplementary Figure S1.

#### Brain tissue volumetry

Results for automated brain tissue volumetry are provided in Table 3 and show a small but consistent variability between super-resolution reconstruction methods, in the order of 1%, except for extra-cerebral CSF, where 2.7% differences were observed between NeSVoR and NiftyMIC. Growth curves for volumetry are provided in Fig. 3 (bottom row) and yield values that generally align with previously estimated centiles [16], except for the cortical gray matter, which was consistently overestimated compared to Kyriakopoulou et al. [16], and underestimated compared to Machado-Rivas et al. [28].

**Table 3:** Statistical analyses for volumetry measurements. Univariate biometry analysis (*N* = 252, df = 2) and multivariate biometry analysis using a t-distributed Generalized Additive Model for Scale and Location (GAMLSS) model

	Univariate analysis					Multivariate analysis		
	Friedman	Post-hoc testing				Super-resolution reconstruction effect		
	*P*-value	Comparison	*P*-value	Effect	Median diff. [cm]	Comparison	Est. effect	*P*-value
**Extra-cerebral CSF**	5.5 × 10^–11^	NeSVoR vs NiftyMIC	> 0.05	–		NeSVoR vs NiftyMIC	−1.84±0.16	**10**^**–16**^
		NeSVoR vs SVRTK	**10**^**–4**^	0.45	−1.8[−12.8,2.3]	NeSVoR vs SVRTK	−0.19±0.18	0.31
		NiftyMIC vs SVRTK	**10**^**–12**^	0.80	2.1[−0.4,10.8]			
**Cortical GM**	4.9 × 10^–14^	NeSVoR vs NiftyMIC	**10**^**–8**^	0.67	0.7[−0.7,2.6]	NeSVoR vs NiftyMIC	−0.68±0.03	**10**^**–16**^
		NeSVoR vs SVRTK	**10**^**–7**^	0.61	0.5[−0.7,2.2]	NeSVoR vs SVRTK	−0.39±0.03	**10**^**–16**^
		NiftyMIC vs SVRTK	**10**^**–3**^	0.36	0.3[−1.5,1.4]			
**Cerbellum**	7.5 × 10^–6^	NeSVoR vs NiftyMIC	> 0.05	–		NeSVoR vs NiftyMIC	−0.04±0.01	**10**^**–12**^
		NeSVoR vs SVRTK	**10**^**–3**^	0.42	0.1[−0.2, 0.3]	NeSVoR vs SVRTK	−0.02±0.01	**10**^**–3**^
		NiftyMIC vs SVRTK	**10**^**–5**^	0.49	0.1[−0.1,0.3]			
**ST**	6.1 × 10^–12^	NeSVoR vs NiftyMIC	**10**^**–10**^	0.71	1.2[−0.7, 4.7]	NeSVoR vs NiftyMIC	−0.84±0.07	**10**^**–16**^
		NeSVoR vs SVRTK	**0.03**	0.29	0.3[−1.5,1.8]	NeSVoR vs SVRTK	−0.43±0.06	**10**^**–11**^
		NiftyMIC vs SVRTK	**10**^**–5**^	0.55	0.5[−0.7,4.0]			
**Lateral ventricles**	1.9 × 10^–7^	NeSVoR vs NiftyMIC	**10**^**–5**^	0.52	0.1[−0.2, 0.3]	NeSVoR vs NiftyMIC	−0.06±0.01	**10**^**–16**^
		NeSVoR vs SVRTK	**0.01**	0.34	0.1[−0.1, 0.3]	NeSVoR vs SVRTK	−0.03±0.01	**10**^**–6**^
		NiftyMIC vs SVRTK	**0.02**	0.39	0.1[−0.2, 0.1]			

*CSF* cerebrospinal fluid, *GM* gray matter, *NeSVoR* Neural Slice-to-Volume Reconstruction, *ST* supratentorial brain tissue, *SVRTK* Slice-to-Volume Reconstruction ToolKit

#### Qualitative feedback on super-resolution reconstruction

In the first qualitative experiment evaluating the presence and visibility of specific anatomical structures on super-resolution reconstructed volumes, clinicians rated most volumes from NeSVoR and NiftyMIC as insufficient for their radiological assessment. While SVRTK images were rated of sufficiently good quality (better quality than NeSVoR, *P* = 0.01), clinicians remained hesitant to use them in a radiological assessment. An excerpt from the results is shown in Table 4, where we see that while all super-resolution reconstruction methods yield good cortical continuity and sharpness, NeSVoR performed poorly on the white matter (layering: SVRTK-NeSVoR = 0.5 (*P* < 0.01), intensity: SVRTK-NeSVoR = 0.63 (*P* = 0.01), NiftyMIC-NeSVoR = 0.54 (*P* < 0.01)) and is blurrier than SVRTK and NiftyMIC (blurriness: SVRTK-NeSVoR = 0.84 (*P* < 0.01), NiftyMIC – NeSVoR = 0.62 (*P* = 0.02)), leading to an overall worse perceived quality (quality: SVRTK-NeSVoR = 0.63 (*P* = 0.01)).

**Table 4:** Subjective structural quality assessment. Scores range between 0 (bad), 1 (acceptable), and 2 (excellent). A *single star* means that the method is statistically significantly better than the worst performing method of the column

Method	Cortex		White matter		Global	
	Continuity	Sharpness	Layering	Intensity	Blurriness	Quality
**NeSVoR**	1.50±0.54	1.50±0.52	0.83±0.56	0.54±0.36	0.58±0.43	0.54±0.58
**NiftyMIC**	1.58±0.50	1.54±0.30	1.17±0.64	1.08±0.59*	1.20±0.52*	0.88±0.70
**SVRTK**	1.50±0.46	1.65±0.37	1.33±0.37*	1.17±0.49*	1.42±0.39*	1.17±0.50*

*NeSVoR* Neural Slice-to-Volume Reconstruction, *SVRTK* Slice-to-Volume Reconstruction ToolKit

**Fig. 4:**
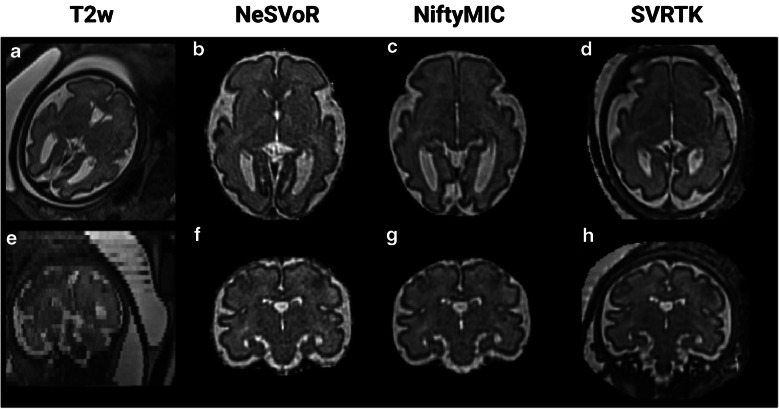
Example of one healthy subject (gestational age = 30w) with axial and coronal views of T2w HASTE acquisitions along with the reconstructed volumes of the best rated quality (global subjective quality = 1.54). **a**–**d** Axial view of one input T2w stack (**a**), the reconstruction using Neural Slice-to-Volume Reconstruction (NeSVoR) (**b**), NiftyMIC (**c**), and Slice-to-volume reconstruction toolkit (SVRTK) (**d**). **e**–**h** Coronal view of one input T2w stack (**e**), the reconstruction using NeSVoR (**f**), NiftyMIC (**g**), and SVRTK (**h**)

**Fig. 5:**
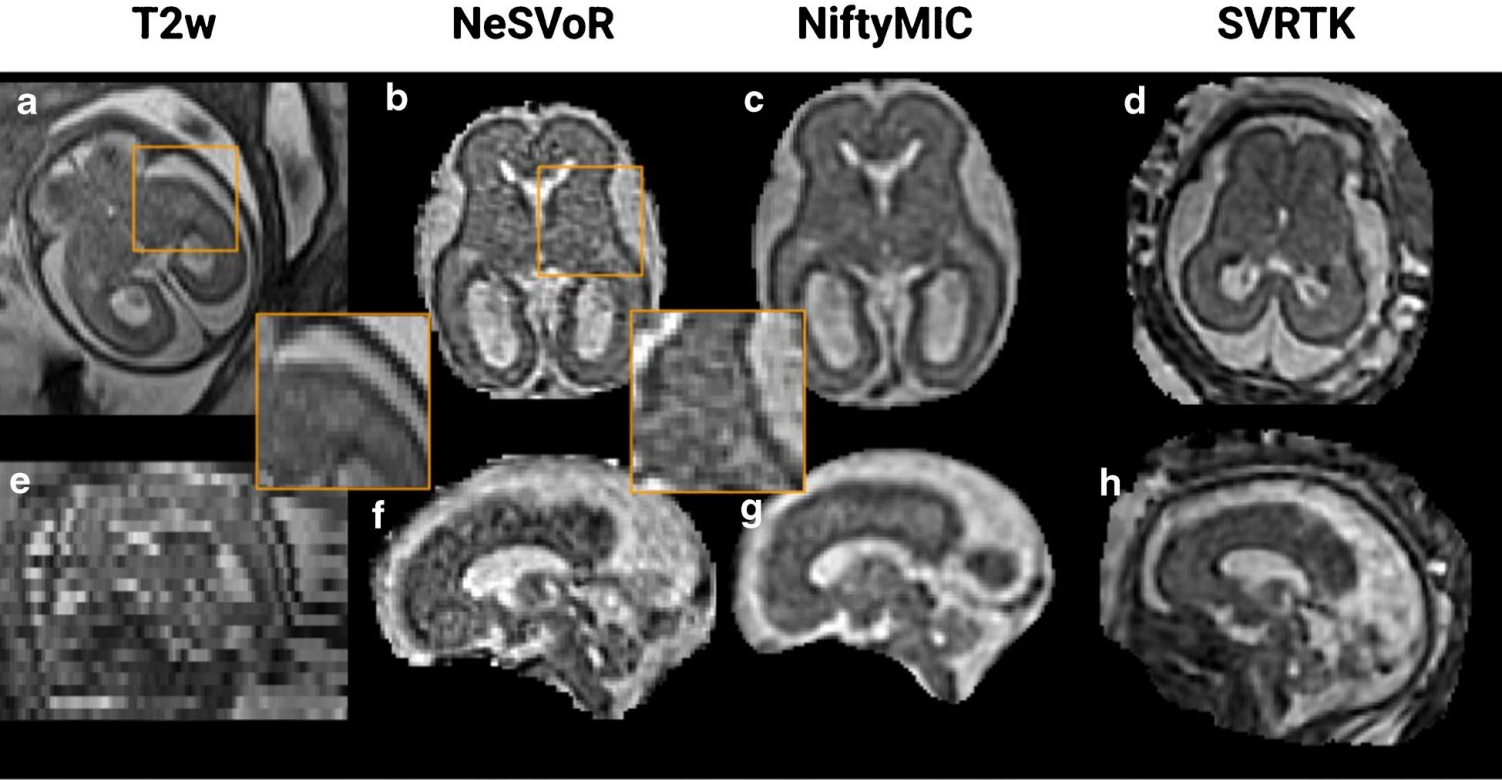
Example of one healthy subject (gestational age = 26w) with axial and coronal views of T2w HASTE acquisitions along with the reconstructed volumes of the worst rated quality (global subjective quality = 0). **a**–**d** Axial view of one input T2w stack (**a**), the reconstruction using Neural Slice-to-Volume Reconstruction (NeSVoR) (**b**), NiftyMIC (**c**), and Slice-to-Volume Reconstruction Toolkit (SVRTK) (**d**). **e**–**h** Coronal view of one input T2w stack (**e**), the reconstruction using NeSVoR (**f**), NiftyMIC (**g**), and SVRTK (**h**)

Additional results on the corpus callosum, ventricles, internal capsule, and posterior fossa are available in the supplementary material. Two examples of reconstructions are shown in Fig. 4 and Fig. 5, where the best and worst rated super-resolution reconstructed volumes are presented respectively, along with the acquired low-resolution stacks in the axial and coronal orientations. Overall, NeSVoR was often graded lower than SVRTK and NiftyMIC due to alterations introduced by the method in the white matter homogeneity and intensity (Fig. 4, subject 1). On the other hand, the best rated volume (Fig. 4, subject 2 with SVRTK) has a very clear white matter, with a marked contrast between the white matter and the basal ganglia.

In the second experiment, in Table 5, the raters ranked the different super-resolution reconstructed volumes between each other, and the low-resolution stacks. The results showed that the NeSVoR reconstructions were consistently rated lower than NiftyMIC and SVRTK, with NiftyMIC rated best in this experiment (super-resolution reconstruction ranking: NiftyMIC-NeSVoR = 0.86 (*P*<0.01)). When compared to the low-resolution stacks, there was no unanimous preference for super-resolution reconstructed volumes over low-resolution images. Experts noted that most of the NiftyMIC and SVRTK volumes were considered usable as low-resolution images but were rather hesitant in using NeSVoR instead of the low-resolution images for their evaluation.

**Table 5:** Qualitative comparison between super-resolution reconstruction (super-resolution reconstruction) and low-resolution (low-resolution). Scores range from 0 to 2, the *first column* reflects a ranking, the *second* refers to whether the clinician would use super-resolution reconstruction instead of low-resolution volumes (choose only one), and the *last column* refers to whether the super-resolution reconstruction was judged more suited for their clinical examination than low-resolution. A score of 1 means that super-resolution reconstruction is as useful as low-resolution

Method	Super-resolution reconstruction ranking	Super-resolution reconstruction *instead* of low-resolution?	Super-resolution reconstruction *better* than low-resolution?
NeSVoR	0.58±0.52	0.79±0.52	0.79±0.72
NiftyMIC	1.46±0.72*	1.21±0.72	1.17±0.63
SVRTK	1.17±0.71	1.08±0.76	1.08±0.78

*NeSVoR* Neural Slice-to-Volume Reconstruction, *SVRTK* Slice-to-Volume Reconstruction ToolKit

## Discussion

Today, advanced image processing techniques such as motion estimation and super-resolution reconstruction allow us to freely navigate in 3-D into the fetal brain to extract quantitative measurements. The aim of our study was to assess whether different state-of-the-art super-resolution reconstruction methods induced systematic biases when reconstructed volumes are used for biometric and volumetric analyses. Results from multi-centric, multi-scanner acquisitions show statistically significant differences in 2-D biometry across super-resolution reconstruction methods, with differences consistently remaining below the voxel width (0.8 mm). On 3-D volumetric measurements, trends are similar, with deviations in the order of 1% (2.5% for extra-cerebral CSF, due to different ways of cropping the brain across super-resolution reconstruction methods). While small, the deviations in volumetry are systematic and might be a concern for future fine-grained analyses. Larger deviations from reference growth curves were observed for the cortical gray matter, where even results from Kyriakopoulou et al. [16] and Machado-Rivas et al. [28] exhibited large variations. This is likely due to differences in reconstruction and segmentation protocols between these two works as well as the data used to train the BOUNTI model [27], as variations in the manual delineation of cortical GM are notoriously hard to control [32].

Our work supplements the study of Ciceri et al. [22], who showed in a more restricted setting (20–21 weeks, mono-centric) the consistency of the measurements done on two super-resolution reconstruction methods. Our results are reassuring towards using super-resolution reconstructed volumes in clinical practice or leveraging and comparing results from different studies: even if different super-resolution reconstruction methods were to be deployed in clinical practice or used in multi-centric studies, biometric and volumetric measurements would remain consistent across sites, thus opening the door to new biomarkers, which cannot be obtained from US or low-resolution stacks. These results are shown to be consistent for data acquired at different magnetic field strength (1.5 T and 3 T) as well as different T2-weighted data resolution (between 0.55 × 0.55 × 2.8 mm^3^ to 1.12 × 1.12 × 3.3 mm^3^) and number of stacks (between 3 and more than 12 stacks).

In addition, while super-resolution reconstruction could be readily used for quantitative measurements, challenges remain due to the differences introduced by super-resolution reconstruction methods (textured noise, intensity variations), which can appear depending on the original resolution settings. In our experiments, this is particularly pronounced in the case of NeSVoR. Therefore, training physicians to distinguish between SR reconstruction artifacts and structural alterations would be paramount when making super-resolution reconstruction widely available. Nevertheless, clinicians generally agreed on the benefits of having *both* low-resolution and super-resolution reconstructed volumes available. This could help in detecting cortical malformations, as the gyrification is more clearly visible on super-resolution reconstruction data since navigating in 3-D in super-resolution reconstruction data helps to reduce ambiguities caused by the uncontrolled sampling with 2-D slices with low-resolution stacks.

This work also shows that the true benefits of super-resolution reconstruction would be revealed for biometric measurements of structure that require precise anatomical orientation. This is the case for median structures like the length of the corpus callosum or the height of the vermis.

Nevertheless, despite this multi-centric and multi-rater study, our work should be further extended to include a holistic evaluation of the reconstructed volumes, notably including their quality and their ability to reconstruct pathological subjects. This would be necessary to truly assess the potential of these reconstruction methods in clinical settings.

Finally, we believe that our results will contribute to building large, comprehensive normative models [16] to characterize normal fetal neurodevelopment more thoroughly. Although most existing datasets are not publicly available (aside from the fetal dHCP), and have been processed with different super-resolution reconstruction pipelines, our results suggest that it should still be possible to integrate the derived biometric and volumetric measurements within a unified framework. This approach is particularly compelling as it bypasses the need to share sensitive data by relying only on highly aggregated, derived measurements. Exciting future opportunities also lie in the use of more advanced biomarkers based, such as those based on cortical folding [33, 34].

## Conclusion

Overall, our study indicates that, when comparable 3-D SR volumes of sufficient quality are achieved, the choice of super-resolution reconstruction method does not introduce large systematic biases in 2-D or 3-D measurements. This will allow to build larger cohorts by pooling subjects processed using different super-resolution reconstruction methods, with good data quality, to study in greater depth prenatal neurodevelopment using advanced biomarkers.

## Supplementary Information

Below is the link to the electronic supplementary material.

Supplementary File 1 (DOCX 662 KB)

## Data Availability

The data used in this study is not open access due to patient privacy reasons. Data can be made available upon reasonable request to M.B.C.
