# Supplementary material for: Biometry and volumetry in multi-centric fetal brain magnetic resonance imaging: assessing the bias of super-resolution reconstruction
Source: Pediatr Radiol. 2025 Aug 8;55(10):2064–75. doi: 10.1007/s00247-025-06347-7 (PMC12513972; doi:10.1007/s00247-025-06347-7)
Supplement: Supplementary file 1 — (DOCX 662 KB) [file 247_2025_6347_MOESM1_ESM.docx]

# Supplementary material

#### Intra-rater reliability between low-resolution and super-resolution reconstruction biometry measurements

**Materials and methods.** Intra-rater reliability was evaluated using Lin’s Concordance Correlation Coefficient^33^.

**Results.** In Table S1, intra-rater reliability is reported for the three raters considered. CCC is very high for most structures (above 0.9) indicating very strong reliability. The lowest scores (although still high) are obtained for median structures (length of corpus callosum and height of the vermis). There is no major concern that a given Super-resolution reconstruction method would lead to a decrease in agreement between the Super-resolution reconstruction and LR. Figure S1 provides a visual comparison with the Pearson correlation coefficient and shows clearly that LCC and HV have more scattered measures compared to bBIP, sBIP and TCD. Moreover, some bias in the measurements can be observed from I.V. and M.K. in the LCC, and N.G. in the HV. This is not surprising given that obtaining precise planes for measurements is challenging in LR stacks.

|  | **I.V.** | | | **M.K.** | | | **N.G.** | | |
| --- | --- | --- | --- | --- | --- | --- | --- | --- | --- |
|  | NeSVoR | NiftyMIC | SVRTK | NeSVoR | NiftyMIC | SVRTK | NeSVoR | NiftyMIC | SVRTK |
| **LCC** | 0.73 | 0.65 | 0.69 | 0.87 | 0.86 | 0.86 | 0.93 | 0.92 | 0.92 |
| **HV** | 0.91 | 0.91 | 0.90 | 0.92 | 0.92 | 0.93 | 0.87 | 0.89 | 0.90 |
| **bBIP** | 0.99 | 0.99 | 0.99 | 0.99 | 0.99 | 0.98 | 0.98 | 0.98 | 0.98 |
| **sBIP** | 0.98 | 0.99 | 0.98 | 0.99 | 0.99 | 0.99 | 0.99 | 0.99 | 0.99 |
| **TCD** | 0.98 | 0.98 | 0.97 | 0.99 | 0.99 | 0.99 | 0.97 | 0.98 | 0.98 |

**Table S1** Lin’s Concordance Correlation Coefficient (CCC) between the LR and SR measurements for each rater. This supplements the results presented in Figure 3. Measurements with CCC below 0.9 are highlighted in blue.
*bBIP* brain biparietal diameters, *HV* height of the vermis, *LCC* length of the corpus callosum, *sBIP* skull biparietal diameters, *TCD* transverse cerebellar diameter


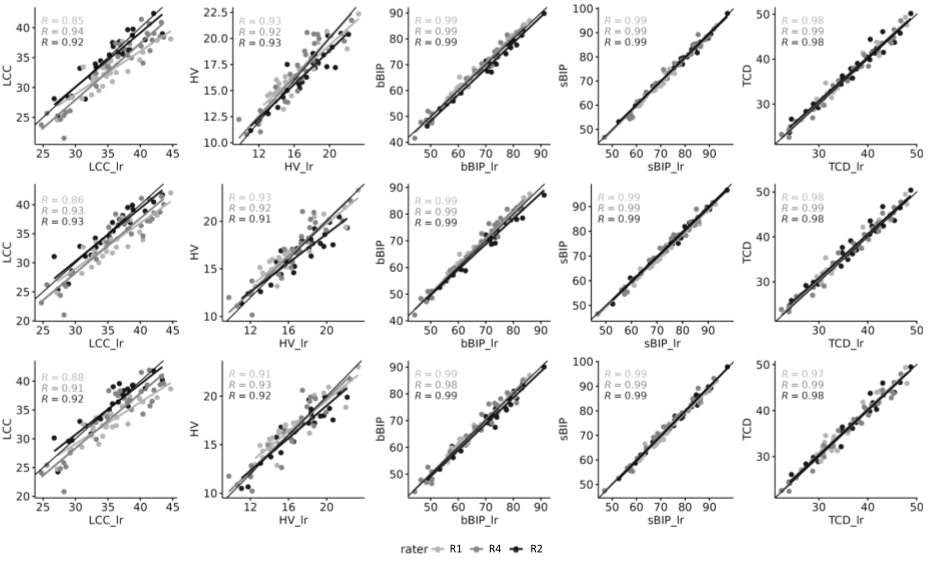
**Fig. S1** Linear regression between the LR and SR measurements for each rater.

*bBIP* brain biparietal diameters, *HV* height of the vermis, *LCC* length of the corpus callosum, *sBIP* skull biparietal diameters, *TCD* transverse cerebellar diameter

#### Complete statistical results for volumetry and biometry

Tables S2 and S3 contain the univariate and multivariate analyses for the biometry, and Tables S3 and S4 contain the univariate and multivariate analyses for the volumetry experiment.

**Table S2** Statistical analyses for biometry measurements. Univariate analysis N= 252, df =2

|  | **Friedman χ^2^** | **p-value** | **Post-hoc testing** | | | |  |
| --- | --- | --- | --- | --- | --- | --- | --- |
|  |  |  | Comparison | p-value | Eff. size | Median diff. [mm] | Median abs. diff [mm] |
| **LCC** | 6.93 | 0.03 | Non-significant after correction for multiple testing | | | |  |
| **HV** | 0.17 | 0.92 |  | | | |  |
| **bBIP** | 9.24 | 9.8 × 10^-3^ | NeSVoR vs SVRTK | 0.03 | 0.28 | 0.3[-2.4, 3.1] | 0.7[0.2,3.4] |
| **sBIP** | 14.55 | 6.9 × 10^-4^ | NeSVoR vs SVRTK | 3 × 10^-4^ | 0.43 | -0.4[-1.9, 1.1] | 0.7[0.1,2.4] |
|  |  |  | NeSVoR vs NiftyMIC | 0.01 | 0.32 | -0.4[-2.3,1.5] | 0.8[0.1,2.2] |
| **TCD** | 11.31 | 3.5 × 10^-3^ | NeSVoR vs SVRTK | 1 × 10^-3^ | 0.38 | 0.4[-0.9, 1.6] | 0.6[0.1,1.6] |
|  |  |  | NeSVoR vs NiftyMIC | 0.02 | 0.30 | 0.3[-0.9, 1.2] | 0.4[0.03,1.8] |

*bBIP* brain biparietal diameters, *HV* height of the vermis, *LCC* length of the corpus callosum, *sBIP* skull biparietal diameters, *TCD* transverse cerebellar diameter

**Table S3.** Statistical analyses for biometry measurements. Multivariate analysis using a t-distributed GAMLSS model

|  | **Super-resolution reconstruction effect** | | | | **Rater effect** | | | |
| --- | --- | --- | --- | --- | --- | --- | --- | --- |
|  | Comparison | Est. effect [mm] | t-val. | p-value | Comp. | Est. effect [mm] | t-val. | p-value |
| **LCC** | NeSVoR vs NiftyMIC | -0.31±0.10 | -3.04 | **0.003** | I.V. vs M.K. | 1.85±0.11 | -3.04 | **0.003** |
|  | NeSVoR vs SVRTK | -0.06±0.10 | 0.65 | 0.51 | I.V. vs N.G. | 0.54±0.11 | 5.06 | **1.1 × 10^-6^** |
| **HV** | NeSVoR vs NiftyMIC | -0.05±0.07 | -0.75 | 0.45 | I.V. vs M.K. | -0.17±0.07 | -2.43 | **0.01** |
|  | NeSVoR vs SVRTK | -0.08±0.07 | -1.28 | 0.20 | I.V. vs N.G. | -0.99±0.07 | -14.06 | **< 2 × 10^-16^** |
| **bBIP** | NeSVoR vs NiftyMIC | -0.25±0.14 | -1.74 | 0.08 | I.V. vs M.K. | 0.56±0.15 | 3.84 | **1.7 × 10^-4^** |
|  | NeSVoR vs SVRTK | -0.35±0.14 | -2.48 | **0.01** | I.V. vs N.G. | -1.15±0.15 | -7.75 | **9 × 10^-13^** |
| **sBIP** | NeSVoR vs NiftyMIC | 0.38±0.09 | 4.04 | **8.2 × 10^-5^** | I.V. vs M.K. | 1.82±0.09 | 19.1 | **< 2 × 10^-16^** |
|  | NeSVoR vs SVRTK | 0.43±0.09 | 4.63 | **7.3 × 10^-6^** | I.V. vs N.G. | 0.32±0.09 | 4.04 | **0.001** |
| **TCD** | NeSVoR vs NiftyMIC | -0.22±0.07 | -3.33 | **0.001** | I.V. vs M.K. | 0.63±0.07 | 9.23 | **< 2 × 10^-16^** |
|  | NeSVoR vs SVRTK | -0.35±0.07 | -5.29 | **3.9 × 10^-7^** | I.V. vs N.G. | -0.63±0.07 | -9.06 | **5 × 10^-16^** |

*bBIP* brain biparietal diameters, *HV* height of the vermis, *LCC* length of the corpus callosum;,*sBIP* skull biparietal diameters, *TCD* transverse cerebellar diameter

**Table S4** Statistical analyses for volumetry measurements. Univariate analysis (N= 252, df =2)

|  | **Friedman χ^2^** | **p-value** | **Post-hoc testing** | | | |
| --- | --- | --- | --- | --- | --- | --- |
|  |  |  | Comparison | p-value | Eff. size | Median diff. [cm^3^] |
| **Extra-cerebral CSF** | 47.21 | 5.5×10^-11^ | NeSVoR vs SVRTK | 1×10^-4^ | 0.45 | -1.82[-12.83,2.28] |
|  |  |  | NiftyMIC vs SVRTK | 7×10^-13^ | 0.80 | 2.11[-0.35,10.75] |
| **Cortical GM** | 61.31 | 4.9×10^-14^ | NeSVoR vs NiftyMIC | 3×10^-9^ | 0.67 | 0.66[-0.74,2.58] |
|  |  |  | NeSVoR vs SVRTK | 7×10^-8^ | 0.61 | 0.46[-0.69,2.23] |
|  |  |  | NiftyMIC vs SVRTK | 0.003 | 0.36 | 0.30[-1.54,1.40] |
| **Cerebellum** | 23.60 | 7.5 × 10^-6^ | NeSVoR vs SVRTK | 3×10^-4^ | 0.42 | 0.06[-0.16, 0.32] |
|  |  |  | NiftyMIC vs SVRTK | 2×10^-5^ | 0.49 | 0.04[-0.10,0.33] |
| **ST** | 51.63 | 6.1 × 10^-12^ | NeSVoR vs NiftyMIC | 3×10^-10^ | 0.71 | 1.16[-0.69, 4.68] |
|  |  |  | NeSVoR vs SVRTK | 0.03 | 0.29 | 0.34[-1.45,1.84] |
|  |  |  | NiftyMIC vs SVRTK | 1×10^-6^ | 0.55 | 0.48[-0.69,3.95] |
| **Lateral ventricles** | 30.93 | 1.9 × 10^-7^ | NeSVoR vs NiftyMIC | 7×10^-6^ | 0.52 | 0.07[-0.18, 0.27] |
|  |  |  | NeSVoR vs SVRTK | 0.005 | 0.34 | 0.05[-0.14, 0.25] |
|  |  |  | NiftyMIC vs SVRTK | 0.02 | 0.39 | 0.05[-0.22, 0.12] |

*CSF* cerebrospinal fluid, *GM* gray matter, *ST* supratentorial brain tissue

**Table S5** Statistical analyses for volumetry measurements. Multivariate analysis using a t-distributed GAMLSS model

|  | **Super-resolution reconstruction effect** | | | |
| --- | --- | --- | --- | --- |
|  | Comparison | Est. Effect [cm^3^] | t-val. | p-value |
| **Extra-cerebral CSF** | NeSVoR vs NiftyMIC | -1.84±0.16 | -11.32 | **< 2 × 10^-16^** |
|  | NeSVoR vs SVRTK | -0.18±0.18 | -1.07 | 0.31 |
| **Cortical GM** | NeSVoR vs NiftyMIC | -0.68±0.03 | -19.87 | **< 2 × 10^-16^** |
|  | NeSVoR vs SVRTK | -0.39±0.03 | -11.42 | **< 2 × 10^-16^** |
| **Cerebellum** | NeSVoR vs NiftyMIC | -0.04±0.01 | -8.15 | **9 × 10^-14^** |
|  | NeSVoR vs SVRTK | -0.02±0.01 | -3.33 | **0.001** |
| **ST** | NeSVoR vs NiftyMIC | -0.84±0.07 | -11.88 | **< 2 × 10^-16^** |
|  | NeSVoR vs SVRTK | -0.43±0.06 | -7.40 | **8 × 10^-12^** |
| **Lateral Ventricles** | NeSVoR vs NiftyMIC | -0.06±0.01 | -11.81 | **< 2 × 10^-16^** |
|  | NeSVoR vs SVRTK | -0.03±0.01 | -5.11 | **9 × 10^-7^** |

*CSF* cerebrospinal fluid, *GM* gray matter, *ST* supratentorial brain tissue

Single-site multi-rater analysis

As the data were rated twice at H3, this allowed us to carry out a more in-depth, single site analysis, removing potential confounders introduced by the nested design of the study. Tables S6, S7 and S8 respectively show the intra- and inter-rater reliability, the univariate biometric analysis and the multivariate analysis. The results are in line with the ones in the main paper, except that in this mono-centric evaluation, the effect of Super-resolution reconstruction is non-significant (the effect size remains the same).

The only additional result is the inter-rater reliability between A.Ma. and N.G., which remains very high overall, although it is slightly lower on median structures, especially in LR vermis height.

**Table S6** Intra and inter-rater reliability. Intra-rater reliability was evaluated using Lin’s Concordance Correlation Coefficient (CC) and inter-rater reliability was evaluated using two-way Intraclass Correlation Coefficient (ICC)

|  | **Intra-rater reliability (LR-Super-resolution reconstruction)** | | | | | | **Inter-rater reliability** | | | |
| --- | --- | --- | --- | --- | --- | --- | --- | --- | --- | --- |
|  | **A.Ma.** | | | **N.G.** | | |  |  |  |  |
|  | NeSVoR | NiftyMIC | SVRTK | NeSVoR | NiftyMIC | SVRTK | LR | NeSVoR | NiftyMIC | SVRTK |
| **LCC** | 0.95 | 0.93 | 0.95 | 0.93 | 0.92 | 0.92 | 0.96 | 0.96 | 0.97 | 0.93 |
| **HV** | 0.97 | 0.98 | 0.95 | 0.87 | 0.90 | 0.90 | 0.89 | 0.95 | 0.95 | 0.94 |
| **bBIP** | 0.99 | 0.99 | 0.99 | 0.98 | 0.98 | 0.98 | 0.99 | 0.99 | 0.99 | 0.99 |
| **sBIP** | 0.99 | 1.00 | 1.00 | 0.99 | 0.99 | 0.99 | 1.00 | 0.99 | 0.99 | 1.00 |
| **TCD** | 0.99 | 0.99 | 0.99 | 0.97 | 0.98 | 0.98 | 0.98 | 0.99 | 0.99 | 0.99 |

*bBIP* brain biparietal diameters, *HV* height of the vermis, *LCC* length of the corpus callosum, *sBIP* skull biparietal diameters, *TCD* transverse cerebellar diameter

**Table S7.** Univariate analysis – Single site and two raters - N=156, df =2. A Kruskal-Wallis test was chosen as Friedman test does not allow for replicated measurements.

|  | **Kruskal-Wallis χ^2^** | **p-value** |
| --- | --- | --- |
| **LCC** | 0.26 | 0.88 |
| **HV** | 0.21 | 0.90 |
| **bBIP** | 0.02 | 0.99 |
| **sBIP** | 0.10 | 0.95 |
| **TCD** | 0.16 | 0.92 |

All median differences are below the voxel resolution (0.8mm isotropic)

bBIP brain biparietal diameters, HV height of the vermis, LCC length of the corpus callosum, sBIP skull biparietal diameters, TCD transverse cerebellar diameter

**Table S8** Multivariate analysis – Single site and two raters – t-distributed GAMLSS model

|  | **Super-resolution reconstruction effect** | | | | | **Rater effect** | | | | |
| --- | --- | --- | --- | --- | --- | --- | --- | --- | --- | --- |
|  | Comparison | Est. effect | t-val. | p-value | | | Comp. | Est. effect | t-val. | p-value |
| **LCC** | NeSVoR vs NiftyMIC | -0.26±0.14 | -1.82 | | 0.07 | A.M.. vs N.G. | | 0.61±0.12 | 5.26 | **6.1 × 10^-7^** |
|  | NeSVoR vs SVRTK | 0.04±0.14 | 0.29 | | 0.77 |  |  |  |  |  |
| **HV** | NeSVoR vs NiftyMIC | -0.03±0.10 | -0.35 | | 0.73 | A.M. vs N.G. | | -0.34±0.07 | -4.29 | **3.5× 10^-5^** |
|  | NeSVoR vs SVRTK | 0.07±0.10 | 0.79 | | 0.42 |  |  |  |  |  |
| **bBIP** | NeSVoR vs NiftyMIC | -0.13±0.22 | -0.57 | | 0.57 | A.M. vs N.G. | | -0.46±0.18 | -2.26 | **0.01** |
|  | NeSVoR vs SVRTK | -0.04±0.22 | 0.16 | | 0.87 |  |  |  |  |  |
| **sBIP** | NeSVoR vs NiftyMIC | 0.36±0.14 | 2.49 | | **0.01** | A.M. vs N.G. | | -0.41±0.12 | -3.53 | **5.9 × 10^-4^** |
|  | NeSVoR vs SVRTK | 0.32±0.14 | 2.25 | | **0.03** |  |  |  |  |  |
| **TCD** | NeSVoR vs NiftyMIC | -0.16±0.08 | -1.92 | | 0.06 | A.M. vs N.G. | | -0.57±0.07 | -6.05 | **1.6 × 10^-8^** |
|  | NeSVoR vs SVRTK | -0.37±0.08 | -4.55 | | **1.3 × 10^-5^** |  |  |  |  |  |

*bBIP* brain biparietal diameters, *HV* height of the vermis, *LCC* length of the corpus callosum, *sBIP* skull
biparietal diameters, *TCD* transverse cerebellar diameter

#### Rater-wise, super-resolution reconstruction-wise regression predictions

In Figure S2, we present a visual representation of the fits obtained using the data from different raters and the different Super-resolution reconstruction methods. It shows visually how more variability in the prediction originates from the rater rather than the Super-resolution reconstruction method.


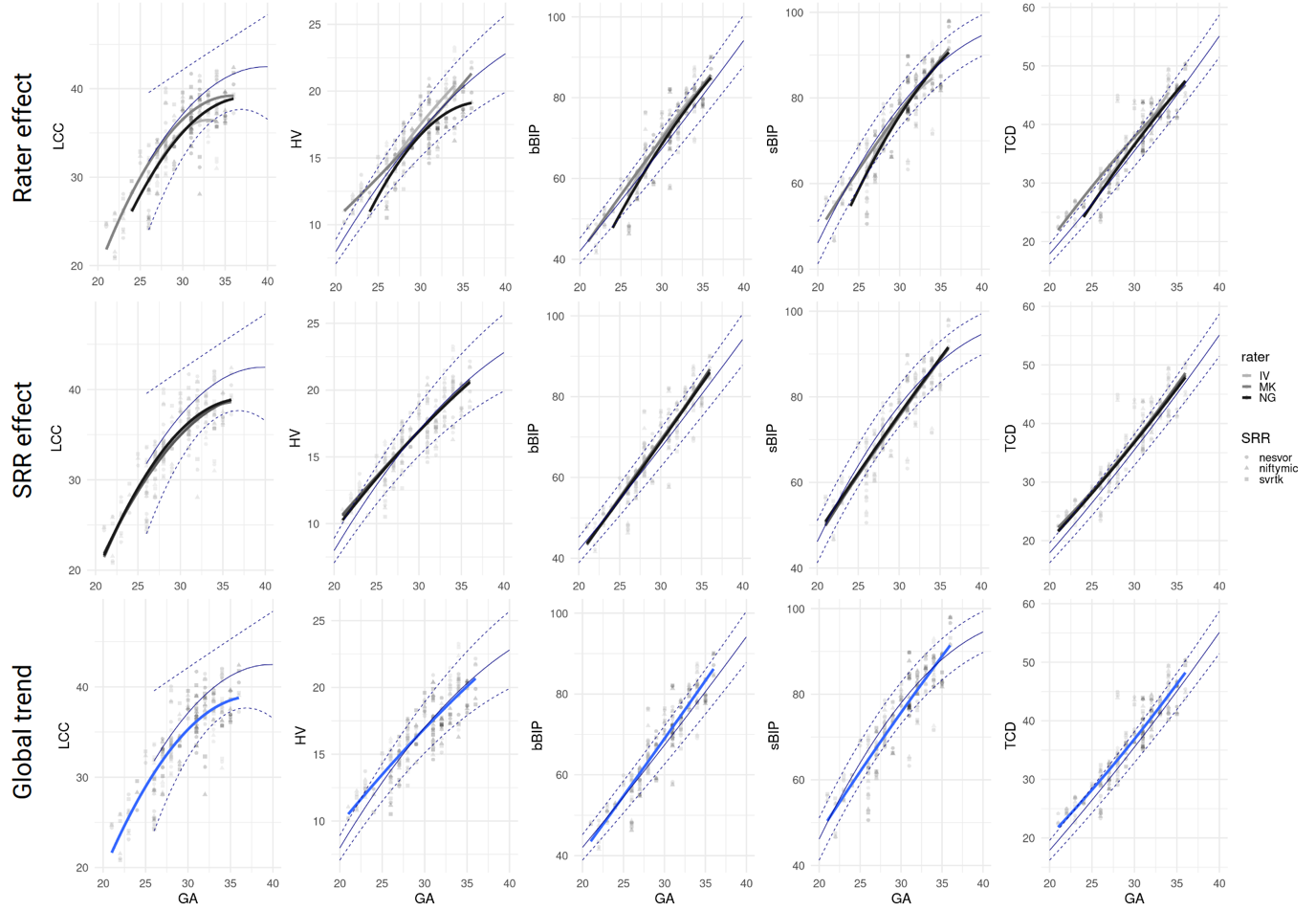
**Fig. S2** Quadratic fit split by rater (first row), by Super-resolution reconstruction method (second row) and global trend (third row). This visually illustrates the sources of variability in the fitting from different sources

*bBIP* brain biparietal diameters, *HV* height of the vermis, *LCC* length of the corpus callosum, *sBIP* skull biparietal diameters, *TCD* transverse cerebellar diameter

Additional results of the subjective rating experiment

**Table S9** Details of the qualitative ratings asked to the raters in the first stage of the subjective evaluation

| **CORTEX** | |
| --- | --- |
| 0 (much broken cortical plate), 1 (some broken), 2 (always visible) | **Continuity** |
| 0 (overall blurry cortex), 1 (blurry at some areas), 2 (sharp and good cortical contrast) | **Sharpness** |
| Does the folding pattern correspond to the estimated GA? 0 (no) 1(yes) | **Folding pattern** |
| **WHITE MATTER** | |
| *Layering appearance visible and according to GA:*  0 (not visible), 1 (partially visible), 2 (perfectly visible) | **Layering** |
| *Overall appearance of WM intensity:* 0 (poor quality, geometric artifacts like lines, dots, pixelization, checkerboard, etc.), 1 (partially unusual appearance), 2 (looks good as clinical series) | **Intensity** |
| **CORPUS CALLOSUM (CC)** | |
| 0 (overall blurry CC), 1 (blurry in some regions), 2 (sharp and good CC intensity contrast) | **Sharpness** |
| *Thickness appears as expected:* 0 (no), 1 (yes) | **Thickness** |
| *Confidence of distinguishing the subsegments of the CC:*  0 (not visible), 1 (somewhat confident), 2 (highly confident) | **Rostrum** |
| *Confidence of distinguishing the subsegments of the CC:*  0 (not visible), 1 (somewhat confident), 2 (highly confident) | **Genu** |
| *Confidence of distinguishing the subsegments of the CC:*  0 (not visible), 1 (somewhat confident), 2 (highly confident) | **Body** |
| *Confidence of distinguishing the subsegments of the CC:*  0 (not visible), 1 (somewhat confident), 2 (highly confident) | **Splenium** |
| *Confidence of distinguishing the subsegments of the CC:*  0 (not visible), 1 (somewhat confident), 2 (highly confident) | **Total length of CC** |
| **VENTRICLES** | |
| Structure is 0 (incompatible with age), 1 (compatible with age) | **Germinal Matrix & Ependyma** |
| Structure is 0 (absent) 1(present) | **Cavum septum pellucidum leaves** |
| Ventricular wall regularity: 0 (all irregular), 1 (focally irregular), 2 (normal) | **Ventricular wall regularity** |
| **INTERNAL CAPSULE** | |
| *Can you distinguish BG &Thalami from surrounding WM?*  0 (not at all), 1 (partially), 2 (clear distinction) | **Internal capsule** |
| **POSTERIOR FOSSA** | |
| *Is cerebellar foliation visible?*  0 (not at all), 1 (partially), 2 (clear distinction) | **Cerebellar foliation visibility** |
| **OVERALL SUBJECTIVE QUALITY ASSESSMENT** | |
| *Overall perceived blurring of the image:* 0 (multiple areas are blurred),  1 (few areas are blurred), 2 (no visible blurring) | **Blurring** |
| *Overall quality of the image:* 0 (I do not like this image), 1(I think that the quality is acceptable, but I would not use it for radiological assessment),  2 (Excellent image quality, I would like to use it for radiological assessment) | **Subjective quality** |

#### Corpus callosum subjective rating

For the corpus callosum, all methods led to a good perception of sharpness and thickness. On the substructures (Table S10A), there was a consistent ordering in the rating quality for all methods (rostrum – genu – splenium/body), independently of the reconstruction method used. On the ventricles, internal capsule and posterior fossa (Table S10B), there was also a consistent hierarchy of NeSVoR < NiftyMIC < SVRTK.

**Table S10** Subjective structural quality assessment, additional results. **(A)** Assessment of the corpus callosum and the clarity of its substructures on the images. **(B)** Assessment of the ventricles (Is the germinal matrix presence compatible with age; are the cavum septum pellucidum leaves present or absence; is the ventricular wall regular), the internal capsule (Are the basal ganglia (BG) and thalami clearly discernable from the white matter) and the posterior fossa (is the cerebellar foliation clear visible)

| **(A)** | **Corpus callosum** | | | | | | | | | | |
| --- | --- | --- | --- | --- | --- | --- | --- | --- | --- | --- | --- |
|  | Sharpness | Thickness | | Rostrum | | Genu | | Body | Splenium | | Total length CC |
| **NeSVoR** | 1.04±0.69 | 0.92±0.28 | | 0.88±0.90 | | 1.25±0.85 | | 1.46±0.72 | 1.33±0.70 | | 1.29±0.69 |
| **NiftyMIC** | 1.38±0.71 | 0.83±0.38 | | 1.04±0.86 | | 1.42±0.83 | | 1.63±0.58 | 1.67±0.56 | | 1.46±0.66 |
| **SVRTK** | 1.42±0.58 | 0.83±0.38 | | 1.08±0.83 | | 1.79±0.51 | | 1.71±0.46 | 1.67±0.64 | | 1.67±0.48 |
| **(B)** | **Ventricles** | | | | | | **Internal capsule** | | | **Posterior Fossa** | |
|  | Germinal Matrix & Ependyma | | Cavum septum pellucidum leaves | | Ventricular wall regularity | | BG&Thalami visibility | | | Cerebellar foliation visibility | |
| **NeSVoR** | 0.88±0.34 | | 0.83±0.38 | | 1.25±0.79 | | 0.79±0.83 | | | 0.88±0.61 | |
| **NiftyMIC** | 0.83±0.38 | | 0.83±0.38 | | 1.33±0.64 | | 1.08±0.78 | | | 1.00±0.78 | |
| **SVRTK** | 0.83±0.38 | | 0.96±0.20 | | 1.46±0.66 | | 1.13±0.80 | | | 1.21±0.78 | |

*NeSVoR* Neural Slice-to-Volume Reconstruction, *SVRTK*, Slice-to-Volume Reconstruction ToolKit


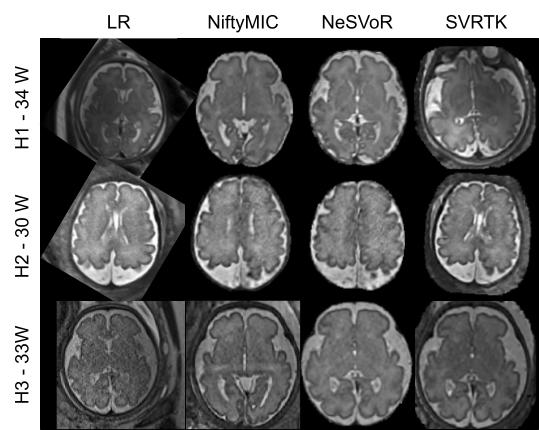


**Fig. S3** Subjects from the three different hospitals reconstructed using the different methods presented in the work. The image from H1 was acquired at 3T, from H2 at 1.5T and from H3 at 1.5 T.
*NeSVoR* Neural Slice-to-Volume Reconstruction, *SVRTK*, Slice-to-Volume Reconstruction ToolKit
